# Th17 Cytokines and Barrier Functions

**DOI:** 10.1155/2016/7179214

**Published:** 2016-04-21

**Authors:** Guansong Wang, Musheng Bao, Xiang Zhang, Juraj Majtan, Kong Chen

**Affiliations:** ^1^Institute of Respiratory Diseases, Xinqiao Hospital, Chongqing, China; ^2^MedImmune LLC, 1 MedImmune Way, Gaithersburg, MD 20878, USA; ^3^Lester and Sue Smith Breast Center, Department of Molecular and Cellular Biology, Baylor College of Medicine, Houston, TX 77030, USA; ^4^Institute of Molecular Biology, Slovak Academy of Sciences, Dubravska Cesta 21, 845 51 Bratislava, Slovakia; ^5^Children's Hospital of Pittsburgh of UPMC, University of Pittsburgh Medical Center, PA 15224, USA

IL-17 functions in host defense against extracellular bacterial and fungal infections and contributes to the pathogenesis of various autoimmune inflammatory diseases. IL-17 producing CD4+ T helper cells (Th17) are a major source of IL-17* in vivo* and play a central role in the pathogenesis of autoimmune diseases. Since the discovery of Th17 cells in 2005, there has been rapid progress in our understanding of their roles and plasticity in the immune system and their defects causing genetic diseases. The IL-17 pathway has been shown to be an attractive therapeutic target in many autoimmune and inflammatory disorders. Most Th17 cells were found to reside in the barrier tissues, including respiratory and intestinal tracts as well as skin. Cytokines produced by these Th17 and non-Th17 cells play critical roles in regulating tissue homeostasis and inflammation.

The pathogenic roles of Th17 in Multiple Sclerosis (MS) have been studied extensively in both human and animal models. E. Volpe et al. discuss recent advances in this field, with particular focus on the mechanisms conferring pathogenicity in MS and their potential modulation. G. R. D. Passos et al. reviewed the key findings supporting the relevance of the Th17 pathways in the pathogenesis of MS and Neuromyelitis Optica Spectrum disorders, as well as their potential role as therapeutic targets in the treatment of immune-mediated CNS disorders. The roles of Th17/IL-17 in other autoimmune diseases are also highlighted in this special issue. R. Kugyelka et al. summarized the recent advancements on the role of IL-17, particularly in the different rodent models of Rheumatoid Arthritis. Y. Li et al. summarized current knowledge about Th17 cells and gut microbiota involved in type 1 diabetes and proposed Th17 targeted therapy in children with islet autoimmunity to prevent progression to overt diabetes. After reviewing current literatures on Th17/IL-17 in asthma, X. Yang et al. proposed that Th17/IL-17 may be a key player in neutrophilic asthma. Recent findings on IL-17-driven mechanisms that promote breast cancer progression were thoroughly reviewed by T. Welte and X. H.-F. Zhang and the contradictory roles of IL-17 in cancer were also discussed. Owing to the increasing evidence for pathogenic roles of IL-17 in various diseases, Th17-targeted therapies are being actively investigated. H. Lin et al. thoroughly reviewed recent development and therapeutic potential of targeting Th17 cells with small molecules and small interference RNA. The research tools for interrogating the roles of IL-17 in human diseases are also of interest of many researchers in this field. Interestingly, F. Neves et al. studied IL17A from five lagomorphs and found that* IL17A* sequences of human and European rabbit are more closely related than the sequences of human and mouse, suggesting that European rabbit might be a more suitable animal model for studying human IL-17.

Several human studies in this special issue confirmed animal model findings. B. He et al. analyzed serum cytokine levels in patients with High-Altitude Deacclimatization Syndrome (HADAS) and found that IL-17A levels correlate with disease incidence and severity, indicating that serum levels of IL-17A could serve as a novel predictive index of HADAS. Y. Gong et al. examined a cohort of Ulcerative Colitis patients in a Chinese Han population and found that the levels of IL-17 and Th17 were significantly higher compared to healthy subjects while the frequency of Treg and the serum TGF-b1 were significantly reduced, suggesting that restoration of the Th17/Treg immune balance might have therapeutic potential in UC management. Using primary human nasal epithelial cells, M. Ramezanpour et al. provided data suggesting that Th17 cytokines may contribute to the development of chronic rhinosinusitis by promoting a leaky mucosal barrier. P. B. Linhartova et al. investigated the association of IL17 and IL17F gene polymorphisms with type 1 diabetes mellitus and chronic periodontitis and found the functional relevance of the* IL17A* polymorphism with higher IL-17 secretion in individuals with A allele. A. B. Christensen et al. observed increased* IL17A* mRNA expression in the intestinal epithelium of antiretroviral therapy (ART) suppressed HIV-1 infected individuals after treatment with histone deacetylase (HDAC) inhibitors (panobinostat), suggesting that panobinostat therapy may influence the restoration of mucosal barrier function in these patients.

Although Th17 cells seem to be mostly pathogenic in the setting of autoimmune diseases, the IL-17 pathway is instrumental in host defense against bacterial infections. Two papers in this special issue focus on the role of IL-17 in host defense: Z. Yan et al. summarize the recent advances in understanding the host-pathogen interaction of* A. baumannii* and propose a potential role of the IL-17 pathway in generating a protective immune response. Using a mouse model of septic peritonitis in mice induced by* E. coli*, Y. Ren et al. found a strong correlation of IL-17 production and surface TLR9 expression on neutrophils, suggesting that both molecules can be therapeutic targets for treating sepsis.

We hope that this special issue will not only be useful to the broad readership by providing insights into new and important aspects related to Th17 cytokines and barrier functions in the context of human diseases, but also stimulate novel research ideas and innovated therapeutic strategies in this field.

